# Critical functional lung volumes in neonatal intensive care: evidence and clinical applications

**DOI:** 10.1038/s41390-022-02450-9

**Published:** 2023-01-09

**Authors:** Theodore Dassios

**Affiliations:** 1 Neonatal Intensive Care Centre, King’s College Hospital NHS Foundation Trust, London, UK; 2 Department of Women and Children’s Health, School of Life Course Sciences, Faculty of Life Sciences and Medicine, King’s College London, London, UK

## Abstract

**Abstract:**

Respiratory disease is common in premature and sick newborn infants and can often necessitate the initiation of intensive care. Newborn infants often suffer from conditions that are associated with decreased lung volumes that occur as a result of abnormal or incomplete lung development. Such conditions are prematurity and respiratory distress syndrome, preterm premature rupture of membranes and the ensuing pulmonary hypoplasia and congenital lung anomalies such as congenital diaphragmatic hernia. These diseases have a structural component manifesting with lower lung volumes and a functional component that can present with increased oxygen and ventilatory requirements. The corresponding decreased functional lung volume is possibly responsible for some unfavourable pulmonary outcomes. Some infants are unable to wean off invasive respiratory support and, in extreme cases, unable to sustain independent breathing that can lead to long-term invasive ventilation or subsequent death. The aim of this review is to summarise the available evidence behind the concept of a critical functional lung volume in neonatal intensive care and describe the clinical implications that arise from decreased functional lung volumes in the main high-risk populations of newborn infants.

**Impact:**

- Newborn infants suffer from diseases such as respiratory distress syndrome, pulmonary hypoplasia and congenital diaphragmatic hernia that are associated with a decrease in the total lung volume and impaired lung function.
- Critically decreased functional lung volumes during neonatal care are associated with failure to wean off invasive respiratory support, increased mortality and possibly longer-term respiratory complications.

## Introduction

In the 1996 film “*The English Patient*”, the protagonist Count Almásy (played by the actor Ralph Fiennes), after having been shot down flying across the desert and badly burned, is being nursed in a bombed-out Italian monastery. While he recalls his history in a series of flashbacks, he shows his nurse (played by Juliette Binoche) his fist and says “l have this much lung” and later “that little bit of air in my lungs each day, it gets less and less”. Count Almásy having recited his story eventually dies. This scene describes the concept of the critical lung volume, or that a certain amount of lung parenchyma is required to sustain unassisted breathing and in some cases can determine the survival of the affected individual.

The human respiratory system has evolved to operate mostly at rest and to rapidly mobilise in conditions of acute cardiorespiratory demand.^1^ When at rest, only a fraction of the respiratory capacity is utilised to maintain oxygenation and carbon dioxide clearance but in conditions of intense cardiorespiratory demand, the majority of the cardiorespiratory capacity can be recruited.^1^ The everyday life activities, thus, of a healthy individual do not require the full recruitment of the respiratory reserves but in states of disease or intense exercise a lack of adequate reserves might become apparent. Newborn infants often face diseases that impact on normal lung development and cause a decrease in lung reserves which might impair their ability to sustain independent breathing or to survive. Moreover, immediately after birth even term newborns may not be able to achieve optimal functional lung volumes, which may explain why respiratory distress still occurs in this population.

Premature birth disrupts normal in utero lung development, and is associated with smaller lung volumes and underdeveloped lungs that are lacking adequate surfactant concentrations.^2^ Preterm premature rupture of membranes (pPROM) might also be associated with pulmonary hypoplasia via loss of amniotic fluid and disruption of the pressure development which drives normal lung growth.^3^ Congenital diaphragmatic hernia (CDH) constitutes a distinct condition associated with pulmonary maldevelopment, also associated with smaller lung volumes.^4^

The clinical importance of these conditions, which are associated with decreased functional lung volumes, is that attempts at weaning from invasive ventilation may not always be successful. In extreme conditions, the reduction of the lung parenchyma is such that adequate oxygenation and survival cannot be achieved despite the provision of invasive ventilation. The aim of this report is to review the available evidence on neonatal lung diseases associated with decreased functional lung volumes and to explore whether there is a critical lung volume that is required for unsupported breathing and survival to discharge from neonatal care. We searched PubMed, Scopus and the Cochrane Central trials register up to June 2022 and performed a manual search of references in narrative and systematic reviews.

## Lung volumes of normal-term infants

Post-mortem studies have indicated that the total lung volume determined by water displacement was between 39.3 and 47.3 ml/kg in term neonates without respiratory pathology.^5,6^ Studies of total lung volumes calculated by MRI in foetuses without respiratory pathology have shown that lung volume increased exponentially with gestational age (GA) from 2–8 to 110–125 ml.^7,8^ In a group of 58 foetuses without any subsequent postnatal respiratory pathology, planimetric MRI measurement of total lung volume, produced a regression equation which estimated a crude volume of 58.8 ml at 40 weeks of gestation.^9^ Studies of foetal lung volume measurements using three-dimensional ultrasound have demonstrated that the expected mean volume at 34 weeks was 27.02 ml.^10^ Since maximal volitional manoeuvres cannot be performed in infancy, lung volumes can alternatively be estimated by measurement of the functional residual capacity (FRC).

Measurement of FRC using nitrogen washout is based on washing out the nitrogen from the lungs, while the patient breathes 100% oxygen. The initial alveolar nitrogen concentration and the amount of nitrogen washed out can then be used to calculate the lung volume at the start of washout. Instead of using 100% oxygen to washout resident nitrogen, FRC can also be calculated with a tracer gas such as sulfur hexafluoride (SF6).^11^ Measurement of FRC using helium dilution is based on the equilibration of gas in the lung with a known volume of gas containing helium. The FRC can be calculated since the subject is connected to a spirometry apparatus of a known volume.^12^ The choice of the FRC methodology can impact the results due to inherent physiological differences and the different technique sensitivities to detect structural damage^13^ with the measured FRC being higher with nitrogen versus SF6.^14^

A study that employed simplified nitrogen washout to measure FRC in healthy term infants without respiratory pathology reported the regression equation: FRC (ml) = 20.4 × weight (kg) – 14.8 (correlation coefficient = 0.979), which for a term infant of 3.5 kg would correspond to an FRC of 16.2 ml/kg.^15^ Seminal studies employing nitrogen washout to measure FRC have also reported mean FRC values for a 3.5 kg term infant of 17.7–31.3 ml/kg.^16–18^ The FRC has also been measured in normal infants using helium dilution and produced a prediction equation that would provide an expected FRC of 15.2 ml/kg for a normal-term infant.^19^

## Prematurity

Preterm infants have smaller lungs^20^ which are not fully developed and functionally disadvantaged. Gas exchange is possible during the canalicular and saccular intrauterine stages of development, when alveolar ducts are forming primitive alveolar sacs and capillary beds^21^ but mature alveolarisation starts late in gestation and continues after birth until 2 years of age or even into adolescence according to some studies.^22,23^ Surfactant is synthesised and secreted by type II alveolar epithelial cells, which maturate between 24 and 34 weeks of gestation^21,24^ with surfactant homeostasis not being fully developed until 32–34 weeks.^25^ Surfactant is critical for achieving and maintaining functional lung volume by decreasing alveolar surface tension resulting in lower required pressures to open the alveolus and prevent collapse.

It follows that preterm infant lungs face the double challenge of functional underdevelopment and structurally small size. The mean FRC was measured in 48 preterm infants at a postconceptional age of 36 weeks during unsedated sleep using a modified heliox/nitrogen washout and was 26.9 ml/kg of body weight in regular breathing.^26^ Latzin et al. also reported that FRC measured by multiple breath washout with 4% SF6 at a postconceptional age of 44 weeks was 22.6 ml/kg in term-born infants and marginally higher (23.4 ml/kg) and lower (21.4 ml/kg) in preterm infants with or without bronchopulmonary dysplasia (BPD), respectively.^27^ Furthermore, Emeriaud et al. in a study exploring the variability of the end-expiratory lung volume (equivalent to the FRC) in 18 premature infants of 30–33 weeks of GA measured by inductance plethysmography, reported that FRC was less variable in ventilated infants compared to unsupported spontaneously breathing infants or infants supported with non-invasive modes.^28^

The small and immature lungs of premature infants can influence their ability to sustain independent breathing and wean off invasive ventilation. This is why the lung volumes of premature infants have been assessed, predominantly in relation to whether they can predict successful extubation.^29^ Of note, the timing of measuring the FRC (before or after extubation) might be clinically relevant as FRC has been shown to fluctuate significantly during the process of extubation, with an FRC loss of >10 ml/kg at the time of extubation compared to before extubation. This finding was described in a study of 12 infants of 26–32 weeks of gestation that were extubated to non-invasive respiratory support and whose FRC was measured by electric impedance tomography.^30^ This temporary loss of FRC, interestingly, was soon regained to levels exceeding the ones before extubation with prone positioning for 10 min.^30^

**Fig. 1:**
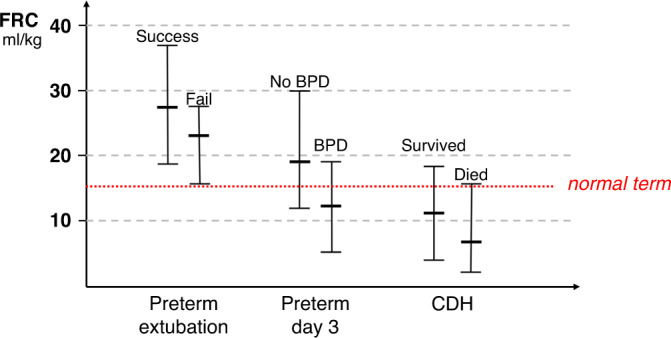
Functional residual capacity (FRC) values adjusted for body weight in premature infants according to extubation outcome^32^ and later development of bronchopulmonary dysplasia^37^ and in infants with congenital diaphragmatic hernia (CDH) according to survival to discharge from neonatal care.^55^ The horizontal lines on the range bars represent the median and range. The dotted line represents the calculated normal FRC in a term infant with a weight of 3.5 kg according to Gerhardt et al.^11^.

**Fig. 2:**
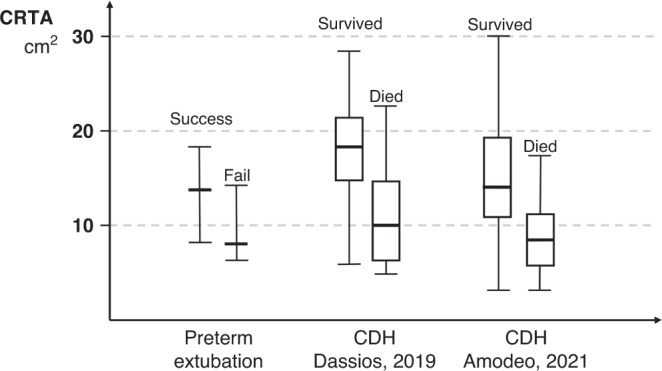
Chest radiographic thoracic area (CRTA) in premature infants according to extubation outcome^34^ and in infants with CDH according to survival to discharge from neonatal care according to Dassios et al.^56^ and Amodeo et al.^61^. Please note that, contrary to Fig. 1, these values are unadjusted for body weight. The central horizontal line represents the median, the horizontal lines of the boxes represent the upper and lower quartile values and the horizontal lines outside the boxes represent the range.

Dimitriou et al. measured the FRC using helium dilution in 20 premature infants with a median GA of 29 weeks and reported that a post-extubation FRC < 26 ml/kg had a 71% sensitivity and 77% specificity in predicting extubation failure (Fig. 1 and Table 1). The authors also demonstrated that the fraction of inspired oxygen (F_I_O_2_) prior to extubation had similar sensitivity and specificity in predicting successful extubation.^31^ This observation might highlight the two structural (FRC) and functional (F_I_O_2_) components required to sustain independent breathing in preterm infants. The same team explored the ability of pre-extubation FRC measurements to predict extubation failure. In a cohort of 30 premature infants with a median GA of 29 weeks, they reported that, unlike post-extubation measurements, the pre-extubation measurements could not differentiate between subsequent successful or failed extubation and that GA had the highest area under the curve (AUC) of all studied parameters in predicting the outcome of extubation.^32^ This observation again possibly highlights the dual functional/maturational nature of the critical lung volume that is required for successful extubation. An indirect two-dimensional estimate of the lung volumes can be obtained by measuring the lung area in chest radiographs and measuring the corresponding chest radiographic thoracic area (CRTA). In a study of 50 infants the CRTA has been shown to correlate significantly (*r* = 0.60, *p* < 0.001) with measurements of FRC taken as a gold standard for measuring lung volumes.^33^ Dimitriou and Greenough measured the CRTA in post-extubation chest radiographs in 20 preterm infants with a median GA of 30 weeks and reported that a CRTA < 8.5 cm^2^ had 100% specificity in predicting extubation failure, but similarly to the FRC studies, the GA and the F_I_O_2_ pre-extubation had a similar prognostic ability (Fig. 2 and Table 1).^34^ The CRTA correlated well with oxygenation indices such as the non-invasive ventilation to perfusion ratio-measured with the oxyhaemoglobin dissociation curve in 22 infants born at a median gestation of 26 weeks with BPD.^35^ A possible limitation of the CRTA method might relate to the phase of breathing during which the radiograph is obtained. Although an effort is made to time the radiograph at end-inspiration, a high breathing rate might make this synchronisation challenging.

**Table 1:** Studies reporting on the ability of different lung volume measurements to predict subsequent neonatal outcomes.

Study	Condition	Number of infants	Gestation (weeks)	Measured parameter	Methodology	Outcome	Predictive ability
Dimitriou, 1996^31^	Prematurity, RDS	20	Median (range): 29 (26–36)	FRC	Helium gas dilution post extubation	Failed extubation	FRC < 26 ml/kg
Dimitriou, 2000^34^	Prematurity, RDS	20	Median (range): 28 (25–33)	CRTA	Chest radiography	Failed extubation	AUC: 0.84 CRTA < 8.55 cm^2^ Sensitivity 57% Specificity 100%
Kavvadia, 2000^32^	Prematurity, RDS	30	Median (range): 29 (25–33)	FRC	Helium gas dilution post extubation	Failed extubation	AUC: 0.81 FRC < 26 ml/kg Sensitivity: 70% Specificity: 70%
Dassios, 2019^36^	Prematurity, RDS	56	Median (IQR): 26 (25–29)	Tidal volume (V_T_)	Measured before extubation	Successful extubation	AUC: 0.786 V_T_ > 4.5 ml Sensitivity: 82% Specificity: 58%
Messerschmidt, 2011^44^	Preterm premature rupture of membranes (pPROM)	40	Mean (SD): 21.9 (2.6) at pPROM Mean (SD): 26 (1.2) at birth	% of expected lung volume	MRI lung volumetry	Death before discharge	Significance of the regression model: 0.014 (OR: 0.927, 95% CI: 0.872–0.985) Sensitivity: 80% Specificity: 86%
Jani, 2007^47^	CDH	354	Median (range) 27 (18–38) at measurement	Foetal lung area to head circumference ratio (LHR)	Transverse section of the foetal chest on 2-dimensional ultrasound	Survival	AUC: 0.761 Sensitivity: 46% for a false positive rate of 10%
Dimitriou, 2000^55^	CDH	25	Median (range) 38 (31–40)	CRTA	Chest radiography within 24 h post operation	Death or oxygen dependency at 28 days	AUC: 0.78
				FRC	Helium gas dilution within 24 h post operation		AUC: 0.71
Dassios, 2019^56^	CDH	84	Median (IQR) 36 (34–39)	CRTA	Chest radiography on day 1 of life	Survival to discharge from neonatal care	AUC: 0.826 CRTA > 12.99 cm^2^ Sensitivity: 85% Specificity:73%
Amodeo, 2021^61^	CDH	77	Mean (SD) 36.6 (2.2)	CRTA	Chest radiography on day 1 of life	Survival during the first year of life	AUC: 0.808 CRTA > 10.87 cm^2^ Sensitivity: 78% Specificity: 75%
Weis, 2021^60^	CDH	255	Median (IQR): 38 (34–40.3)	CRTA	Chest radiography on day 1 of life	Death before discharge	AUC: 0.822 CRTA < 8.06 cm^2^ Sensitivity: 88% Specificity: 69%

*AUC* area under the curve, *CDH* congenital diaphragmatic hernia, *CI* confidence intervals, *CRTA* chest radiographic thoracic area, *FRC* functional residual capacity, *IQR* interquartile range, *LHR* foetal lung-area-to-head circumference ratio, *OR* odds ratio, *pPROM* preterm premature rupture of membranes, *RDS* respiratory distress syndrome, *SD* standard deviation.

In the era of volume-targeted ventilation, the unadjusted-for-body-weight (crude) tidal volume during invasive ventilation has been shown to hold some prognostic capacity in predicting the outcome of extubation. In 56 preterm ventilated infants with a median GA of 26 weeks, unadjusted tidal volumes had an AUC of 0.786 in predicting successful extubation and a tidal volume >4.5 ml could predict successful extubation with 82% sensitivity and 58% specificity.^36^ That study speculated that a certain critical amount of lung parenchyma was more important for successful extubation than the developmental maturation and that the unadjusted tidal volume acted as a composite index that incorporated information on both maturity and respiratory state as larger tidal volumes would be required to achieve adequate oxygenation and carbon dioxide clearance in infants with more severe respiratory disease.^36^ This observation might also explain the apparent discrepancy that the adjusted-for-weight FRC (ml/kg) is larger in preterm than in normal-term infants (Fig. 1), although different methodologies might also contribute.

Decreased lung volumes in preterm infants over the first month of life have been associated with a subsequent development of BPD. A study of 74 infants with a median GA of 30 weeks studied consecutively over the first month of life, demonstrated that FRC measured by helium dilution was lower throughout the whole period in infants that subsequently developed BPD, an effect that was more pronounced in the group of infants that developed moderate/severe BPD.^37^ The median FRC increased from 19.3 ml/kg on day 3 of life to 24.4 ml/kg on day 28 in infants without BPD compared to an increase from 12.9 ml/kg on day 3 to 17.8 ml/kg on day 28 in infants with BPD (Fig. 1).^37^ These differences, however, are not the result of straightforward comparisons. Evidently, BPD is a complex multifactorial disease and cannot be fully explained only by differences in FRC as the BPD group was also more immature and was exposed to a higher oxygen concentration and a longer duration of invasive ventilation.

Whether preterm infant lung volumes can determine outcomes later in life and post discharge from neonatal care remains a matter of contention. Schulzke et al. measured the FRC by multiple breath washout with 5% SF6 in 38 infants with a mean GA of 27.8 weeks at 15–18 months of corrected age and reported that FRC increased by an average of 1.1% for every additional week of gestation and decreased by an average of 1.5% for every additional week of respiratory support.^38^ These findings imply that catch-up lung growth has not been achieved, at least by the age of 15–18 months. Proietti et al. examined whether low lung volumes in preterm infants can predict later respiratory morbidity in early childhood. In a group of 166 preterm infants with a median GA of 29 weeks whose FRC was assessed by multiple breath washout with 4% SF6 at 44 weeks postconceptional age, the FRC measurements could not predict any of the planned outcomes of wheeze, hospitalisation, or home oxygen therapy after one year of age.^39^

## Preterm premature rupture of membranes

pPROM has been associated with the subsequent development of pulmonary hypoplasia with an earlier GA at the time of rupture being associated with more severe hypoplasia.^40^ The European inhaled Nitric Oxide (iNO) registry reported that in the 72 infants with pulmonary hypoplasia who were treated with iNO during a 10-year period, thirty-one infants died, and 19 survivors developed chronic lung disease.^41^ pPROM is often associated with preterm birth and thus the complications might be partly explained by the sequelae of prematurity.^42^ The relationship of low lung volumes with pPROM and premature birth though, is not always straightforward. Story et al. in a study of fourteen women who delivered before 32 weeks of gestation and underwent a foetal MRI reported that foetal lung volumes were smaller in foetuses that delivered preterm, both with and without pPROM compared to term-born controls. These findings suggested that the antenatal aetiology for the smaller lung volumes might also have been associated with premature birth.^43^ A prospective study of 163 pregnancies complicated by pPROM from 15 to 28 weeks of gestation reported an incidence of pulmonary hypoplasia of 12.9% diagnosed by clinical, radiological or pathological criteria. The authors demonstrated that the GA at rupture and the latency period between rupture of membranes and subsequent delivery were independent determinants of perinatal death.^40^ Messerschmidt et al. used foetal MRI to measure the lung volumes of 40 foetuses with pPROM at 16–27 weeks of gestation and reported that the estimated-to-expected lung volume was 73% in non-survivors and 102% in survivors, no survivors with lung volume <60% of expected and that mortality could be predicted by the foetal lung volumes with 80% sensitivity and 86% specificity (Table 1).^44^ Kieffer et al. studied at two years of age 31 infants born before 33 weeks of gestation with pPROM between 14 and 24 weeks and reported that they were hospitalised more often for bronchiolitis during their first 2 years compared to controls matched for GA.^45^ Tanigaki et al. performed MRI lung volumetry in 90 foetuses at 25–39 weeks gestation and reported that the lung volume in foetuses with pulmonary hypoplasia was significantly lower than for foetuses with normal lung development and that abnormal foetal lung volumes by MRI had higher diagnostic accuracy for pulmonary hypoplasia than abnormal ultrasound parameters.^46^

## Congenital diaphragmatic hernia

CDH can serve as a model of how a critical functional lung volume is necessary for independent breathing and subsequent survival. The prognosis of CDH, including survival to discharge, is mainly determined by the degree of pulmonary hypoplasia.^4^ Prenatally pulmonary hypoplasia is most commonly assessed by the two-dimensional lung-area-to-head circumference ratio (LHR). A multicentre study, involving 184 foetuses with isolated, left-sided CDH, reported the LHR at 22–28 weeks of gestation provided a useful prediction of subsequent survival.^47^ The LHR increases exponentially with GA in normal foetuses reflecting normal lung development;^48^ consequently, the assessment of foetal lung volumes in CDH is corrected for the GA-related change in LHR. In this sense, many authors have used a relative ratio where the observed lung measurement is compared to an expected value according to the GA at measurement and have reported that a ratio <0.35 is associated with poor prognosis.^49,50^ A multicentre study of 354 CDH foetuses reported that the LHR increased but the observed-to-expected LHR did not change significantly with GA and that the observed-to-expected LHR could predict survival with an AUC of 0.761 (Table 1).^48^ It should be mentioned that methodologically the LHR is not completely independent of GA and the lung-to-thorax transverse area ratio is possibly less influenced by GA.^51^

Ruano et al. used prenatal three-dimensional ultrasound to estimate foetal lung volume in 8 CDH infants and 25 controls and compared these measurements to post-mortem volume measurements. They reported a high agreement between the two methods with an intra-class correlation coefficient of 0.95–0.99. The average foetal lung volume was 9.66 cm^3^ in CDH and 24.54 cm^3^ in controls.^52^ The same team used sonographically measured foetal lung volumes corrected for body weight in 40 CDH foetuses and reported that this ratio was significantly lower in neonates that died than in those who survived and that a low ratio had 64.5% accuracy in predicting death.^53^ Adaikalam et al. measured the lung volume and density to calculate lung mass using foetal and post-CDH repair MRI scans in 12 infants with left-sided CDH and demonstrated that the ratio of left-to-right lung mass significantly increased in the post-repair MRI compared to the antenatal scans, pointing towards an increased rate of growth in the more hypoplastic ipsilateral lung.^54^ This study though did not report on the performance of the lung measurements to predict survival, as it included only post-repair scans and successful extubation and survival were universally achieved.^54^

Dimitriou et al. measured the CRTA and FRC before and after operative repair in 25 CDH infants treated before the year 2000 and reported that the two indices correlated significantly and that postoperatively, a low CRTA performed well in predicting death or oxygen dependency at 28 days (Fig. 1 and Table 1).^55^ The same study reported that the median FRC and CRTA were lower in infants that died or were oxygen dependent at 28 days (7.4 ml/kg and 12.4 cm^2^, respectively) compared to infants with good outcome (11.6 ml/kg and 18.6 cm^2^, respectively). In a later study that included 84 infants with CDH treated between 2007 and 2017, we also described that CRTA predicted survival to discharge with an AUC of 0.826 and that a CRTA exceeding 12.99 cm^2^ predicted survival to discharge (Fig. 2).^56^ Contrary to some previous studies,^57,58^ in this study following multivariable logistic regression the GA was not related to mortality, probably because the CRTA was not corrected for body weight.^56^ Similarly to premature infants, this implies that a certain uncorrected/crude lung volume might be required to sustain independent breathing irrespective of lung maturation and that the CRTA incorporated information on gestation, as more premature infants would have a lower crude CRTA. Of note, the studies that reported that mortality in CDH was related to GA, included a more premature population (median GA was 34 weeks in Ali et al.^57^ compared to 36.6 weeks in Dassios et al.^56^) and this might impact the ability of the latter study to identify prematurity as a significant contributor to mortality. Although beyond the scope of this review, survival in CDH would also be influenced by parameters not related to lung volumes such as fixed/structural pulmonary arterial hypertension secondary to in utero remodelling of the pulmonary arteries.^59^ Despite these limitations, the ability of CRTA to predict survival has been confirmed in subsequent studies from other surgical perinatal centres (Fig. 2 and Table 1).^60,61^ Although strictly not a volume measurement, a low ventilation-to-perfusion ratio (V_A_/Q) in prematurity is the main determinant of a decreased respiratory surface area^62^ which when critically reduced, can also limit gas exchange and influence survival. We have reported that in CDH, a low V_A_/Q measured by the oxyhaemoglobin dissociation curve during neonatal care was associated with increased mortality and that a V_A_/Q in the first 24 h of life exceeding 0.15 predicted survival to discharge with 84% sensitivity and 88% specificity and an AUC of 0.905.^63^ A markedly low V_A_/Q in CDH infants is predominantly a result of diffusion limitation secondary to marked pulmonary hypoplasia (and low lung volumes) as the perfusion deficit has been reported to remain relatively unchanged during the initial days of intensive care^63^ a phenomenon that possibly persists for the first two decades of life.^64^ The anatomical deadspace during initial resuscitation has also been assessed in 30 CDH infants using volumetric capnography and CDH infants who survived had larger deadspace than those who died. The authors postulated that this was a reflection of less lung hypoplasia as insufficient branching of bronchioles in those with more severe lung hypoplasia would lead to fewer smaller non-conducting airways and hence a lower anatomical deadspace.^65^

## Clinical applicability and suggested further research

As highlighted throughout the manuscript, the estimation of lung volumes can predict with variable accuracy outcomes such as successful extubation and survival while low lung volumes can also be involved in the pathogenesis of BPD and early childhood respiratory morbidity. These outcomes are relevant for providing guidance to the families of the affected infants and can possibly guide the clinicians to predict the appropriate timing of extubation or stepping down of respiratory support.

The clinical utility of FRC to evaluate the current state of lung volume and risk stratification may be limited, as interventional respiratory practices aimed at achieving a larger FRC in the delivery room have not shown improvements in respiratory outcomes. Some animal studies showed promising results in the role of sustained inflation to establish FRC in very preterm infants at birth^66^ but subsequent clinical trials failed to demonstrate any benefit of this intervention compared with standard intermittent positive-pressure ventilation in reducing the risk of BPD or death and the study was stopped early due to higher mortality in the sustained inflation group.^67,68^

Undoubtedly, clinical outcomes in neonatal respiratory disease cannot only be predicted by a single measurement of lung volumes, irrespective of the methodology used to obtain these measurements. Although beyond the scope of this review, reduced pulmonary perfusion, intra- and extra-pulmonary shunting most likely play an important role in determining clinically important lung disease as well as ventilation impairment, ventilation to perfusion mismatch, distorted upper airway anatomy and impairment of the control of breathing. We should also acknowledge that diseases such as BPD and CDH are complex and multifactorial with significant shifts and advancements in our understanding over the past 20 years. It follows that presenting results from studies that are older than this, could affect the generalisability of our conclusions in the current era. The lack of better quality evidence, however, and the rarity of some diseases such as CDH were the reasons why some older studies were retained in this review.

This review focused on prematurity, pPROM and CDH which are relatively common conditions characterised by decreased lung volumes and have been more extensively studied. Other such conditions associated with impaired functional lung volumes in the neonatal period are congenital pulmonary airway malformation, bronchial cysts and congenital emphysema but these conditions are rarer and the literature more limited.

The overall predictive ability of lung volume measurements is arguably moderate and GA and F_I_O_2_ requirement often performed similarly to lung volumes. Precise antenatal and postnatal measurement of lung volumes is methodologically cumbersome and would require elaborate and expensive equipment and would therefore be difficult to implement in the clinical setting. A more accessible approach to lung volume measurements might be the measurement of the CRTA. Clinical outcomes, though, are associated not only with the actual volume, but also with the functional state of the lungs, and it is thus plausible that composite indices that combine lung volume measurement and information on the functional state of the respiratory system might be more successful in predicting neonatal respiratory outcomes. Such functional indices might be the ventilation-to-perfusion ratio,^69^ the alveolar deadspace^70^ and the fraction of inspired oxygen that is required to maintain normoxia. Continuing this line of thought, a combination of structural and functional measurements that are both clinically accessible at the cot-side, could be the optimal cost-benefit option: such an approach could be the CRTA divided by the F_I_O_2_. If the concept is proven in a future study, the results should ideally be replicated in a randomised setting where, for example, the decision to extubate can be based on such composite functional volume indices or standard clinical practice.

## Conclusions

Sick newborn infants that receive intensive care face numerous respiratory conditions that are characterised by critically decreased lung volumes. Such conditions are premature birth and respiratory distress syndrome, pPROM and the ensuing pulmonary hypoplasia and congenital lung anomalies such as CDH. These diseases commonly affect both the total lung volume and the functional integrity of the newborn lungs. Critically decreased functional lung volumes are associated with failure to wean off invasive respiratory support and increased mortality before discharge from neonatal care.
